# The Speculum Applied to the Diagnostic Treatment of the Organic Diseases of the Womb

**Published:** 1837-01

**Authors:** 


### Art. XI.

*The Speculum applied to the Diagnostic Treatment of the Organic Diseases of the Womb: an Inaugural Dissertation presented to the University of Glasgow, for the Degree of Doctor in Medicine.* By John Balbirnie, a.m. *London,* 1836. 8vo. pp. 335.

We agree with Dr. Balbirnie, that the French writers have, within the last few years, added much to our knowledge of the pathology and treatment of uterine diseases; and it must be admitted, too, that upon these subjects *we* have not kept pace with our continental brethren. We have had, indeed, from English writers some valuable monographs upon uterine diseases; but those who are acquainted with the writings of Bayley, Dance, Desormeaux, Boivin, Lisfranc, &c. (to speak only of French authors,) must be well aware how largely their labours have been profited by, although the source from whence the chief information has been derived has not always been very openly acknowledged. For the purpose of exciting the attention of the profession in England to this important part of the pathology of females, Dr. Balbirnie proposes in the present work to "give an exact history of the diseases of the womb; to investigate their causes; to describe their symptoms; to point out the organic alterations on which they depend; to indicate their diagnostic characters; and to discuss the modes of treatment." (*Pref.* p. x.) The materials of the work are confessedly collected from various French writers, and from the practice of the Paris hospitals, where, during two years' study, Dr. B. devoted especial attention to these diseases.

The book commences with an "introductory address on the necessity of exploration by means of the speculum," and the more frequent employment of this instrument is urgently recommended, that we may be enabled to detect the existence of various diseases that must remain undiscovered by any ordinary examination. Dr. Balbirnie's anxiety on this subject, and his zeal for the new mode of exploration, are amazing. "We go forth (he says,) its advocate. We proclaim ourselves *the apostle of the speculum.* With that instrument we link our fate, and by that we will stand or fall." (P. 13.) And, as "the ladies are not obdurate, as their character is to be gentle and easily entreated," (p. 19,) Dr. B. feels persuaded that any difficulties on the score of modesty will easily be conquered, if the physician is firm in insisting on the necessity of such an examination. In the performance of so delicate a duty as the use of the speculum, Dr. B. hopes that, as far as regards ourselves, "all that dignity and that delicacy of feeling which ought to distinguish the medical character will be for us a solemn duty and a solemn observance, never to be infringed, never to be compromised. If at any time (he adds,) the medical character should merge into, and invest itself with all the sanctity of the sacerdotal, it is certainly in such circumstances. He is for the time a high-priest, the alone privileged to enter into a *sanctum sanctorum*, whose recesses are not to be exposed to vulgar gaze, as its revelations are to remain buried in the depths of his own heart." (P. 21.) We join with the author in his "hopes," although we wish he had not adopted so absurd a mode of expressing them. We are by no means inclined to undervalue the practical advantages that are often to be obtained from the use of the instrument of which our author is the "apostle," but we are quite sure that, in this country, practitioners will never attempt, nor our patients ever submit to, the very frequent employment of it which he recommends.

We pass over the historical sketch "of the pathology of the diseases of the womb, from the Father of Medicine downwards," as well as the chapter "on the Womb," in which Dr. B. informs us that "it is in a sort an exterior organ;" and come to the "Causes of the Organic Diseases of the Womb." Engorgement and hypertrophy, with chronic inflammation of the womb, not unfrequently result from obstruction of the menses, and at the natural period of the cessation of the menses various "accidents," *anglice* symptoms, are to be apprehended. But our author must speak for himself. "At the critical age, again, it [menstruation] is, in many cases, no longer a moderate flux, as in former times: they are, in fact, torrents of blood frequently which are determined to the organ, and flow from it. Nervous accidents are awakened by the excessive losses, and by the cessation of a function which, during so many years, had exercised so important an influence on the moral and physical character of woman." (P. 52.) Lisfranc has met with organic alteration of the womb before the age of puberty; and Carron de Villards had a case in which "polypus, with engorgement of the body of the womb, existed in a child seven years old." We are sorry we are not informed how this "engorgement" was discovered in a patient of so early an age. It has been observed that, in very young women, the body of the womb is usually diseased; while, "in those living in sexual communication," the neck is more frequently the part affected.

Long and difficult labours not unfrequently lead both to acute and chronic uterine diseases.

"A remark it is important to make, regarding a very frequent cause of chronic leucorrhœa in females, here suggests itself: viz. the too long continuance of the lochial discharge; when this continues beyond five or six weeks, it is frequently the starting point of this infirmity, which remains long permanent. When it continues longer than a month, there is an indication at once to stop the discharge, by a slight cauterization of the mucous follicles of the orifice of the neck of the uterus; otherwise, there is the risk of a true catarrhal flux being induced." (P. 56.)

"Corpulent women with scanty menstruation is so common a circumstance as hardly to constitute a pathological state; but if the patient is thin and meager, the reverse of plethoric, and with but a scanty menstruation, we may be almost certain that there is something anormal as regards the state of the uterus." (P. 60.)

It would be superfluous to offer any comment upon the absurd and ungrammatical mode of expression here used; but we differ from the opinion expressed, as far as we can unravel it. We agree with the author, however, in admitting that "the *heredity* of certain uterine affections—cancer, for example,—is a point indisputable;" although we cannot admit that there is such a term as "heredity" in the English language. The symptoms of the organic diseases of the womb are briefly described in the third chapter. "The commencement of these diseases is often very slight, and takes place in a manner latent, and almost insensibly." The indiscriminate use of pessaries in all cases of prolapsus uteri, without taking into consideration the pathological state of the organ which has produced it, is very properly condemned.

"We may also rank among the symptoms of disease of the womb, *hysteria.* Much discussion has been agitated in the medical world regarding the seat and pathological anatomy of this disease. For us, the womb is clearly the seat of lesion; and an *irritation* of this organ, which is not so easily precised, whether of inflammatory or nervous nature, is the *point de départ* of the symptoms: and the fact we have already alluded to, of all the phenomena of hysteria being produced artificially at will, by irritating injections into the interior of the womb, is quite conclusive, we think, for this view of its pathology." (P. 73.)

That hysteria very frequently depends upon functional derangement or positive disease of the uterus, as its remote or exciting cause, may be safely granted; but that it *always* arises from the "not so easily *precised*" cause assigned by Dr. B., we can by no means admit; nor do we perceive that his general conclusion is at all strengthened by the fact, (*if* indeed we understand his language,) of "the voluntary determination of all the symptoms of hysteria," from throwing irritating injections into the cavity of the womb. Dr. B. is of opinion that *chlorosis* "has nothing to do with the uterus, in an infinite majority of cases." He contends, and we think correctly, that the uterus is only affected secondarily and sympathetically, as the result of the enfeebled energy of all the functions.*

Under the head of Disorders of Menstruation, the author enumerates "stormy menstruation," which variety, we are told, "is one of those comprised in the dysmenorrhœa of authors." In this "stormy menstruation" the patient suffers great pain, and an organic disease of the uterus may result if the case be neglected; the practitioner is enjoined to ascertain the state of the organ, "in the interval of the menses." "Sometimes the womb is found sound; the pains are then purely nervous. The woman *accuses* something which seems to lift up the belly; she experiences contractions and violent desires." (P. 90.)

The chief object of the sixth chapter is to urge the necessity of using the speculum in cases of mucous discharges from the vagina, that the real nature of the disease may be ascertained. The information afforded

* "A favorite *martial* preparation of M. Blaud, in these cases, has been crowned with much success. Take sulphate of iron and subcarbonate of potash, of each half an ounce; reduce separately these two substances to a fine powder, then mix gradually; add mucilage of gum adragant, q. s. to be pounded strongly, and the mass divided into forty-eight *bols.* His method of administration is as follows:—The first, second, and third days, a pill fasting in the morning, and one in the evening; the fourth, fifth, and sixth days, an additional pill at mid-day; the seventh, eighth, and ninth days, two pills morning and evening; the tenth, eleventh, and twelfth days two additional pills at mid-day; the thirteenth, fourteenth, and fifteenth days, three pills morning and evening; the sixteenth and following days, four pills in the morning, the same number at noon and in the evening." (P. 75.)

by the eyesight, Dr. B. gravely assures us, is much more definite and precise than that afforded by mere "palpation." We do not deny the truth of the assertion, but, unless we are mistaken, this ocular demonstration will not be often submitted to in this country for mere mucous discharges.

Various "Cases illustrative" are detailed, and we must say with very unnecessary minuteness, to show the advantages of cauterizing the cervix uteri with the nitrates of mercury and silver, &c., in cases of leucorrhœa and gonorrhœa. A few remarks are made on ulcerations of the neck of the womb, which, according to the extensive experience of M. Ricord, are found, in cases where there is profuse and obstinate leucorrhœal discharges, to be seated at the orifice of the os tincæ, in nineteen cases out of twenty.

"These ulcerations of the neck of the uterus, though simple in their nature, and never of themselves degenerating into cancer, may end, however, in the destruction of the organ." (P. 137.)

Dr. B.'s description of simple hypertrophy, chronic inflammation, engorgement of the womb, and the simple scirrhous induration, contains a very fair practical sketch of these important diseases. He takes his "cases illustrative" from Duparcque, Tealier, &c.

The chapter on "Cancer of the Womb" is the best in the work. It contains a very good account of the pathology and symptoms of this fearful malady, and terminates with the details of various cases from different French authors. A brief account is given of Polypi and Fibrous Tumours of the Womb. M. Lisfranc, it appears, sometimes twists off the polypus with a pair of long pincers; but "the preferable operation is excision" with curved scissors.

The volume terminates with some remarks on the medical and surgical treatment of organic diseases of the womb. We are told that "M. Lisfranc counts great success in the amputation of uterine necks." We presume that M. Lisfranc will lose no time in proving to his professional brethren that he has "counted" correctly. We will not at present venture to say more upon this very delicate and painful subject, than that M. Lisfranc is imperatively called upon to answer the *very* serious charges that have recently been brought against his veracity by one of his pupils.*

We cannot close our account of Dr. Balbirnie's work without offering a few comments upon the very extraordinary, and in every point of view very objectionable, style in which it is written. To say nothing of the inaccuracies of the quotations from the learned languages, the extracts which we have quoted are quite sufficient to show that Dr. Balbirnie is still more unhappy in his own; and we fear that the following additional specimens of his diction (not a tithe of what we had marked,) will justify us in saying that his fault is not that of mere carelessness, but of laboured affectation and bad taste. For example: "There is a disease the most common—the scourge of society—most felt in its consequences by the men than by the women who *actually carry it*."—"It is leucorrhœa." (P. 5.)—"The patient being removed from the menstrual period." (P.

* Maladies de l'Uterus d'après les Leçons Cliniques de M. Lisfranc. Paris, 1836.—8vo.

131.)—"The *unsuccess* of all that she had been able to do." (P. 166.) —"Her health, which was in the most flourishing state, and accompanied with a superb carnation." (P. 243.)—Every pain and every inflammation are called "lively."—"Discharges of blood from the uterus may occur in women *still menstruated.*" (P. 39.)—"We begin with a *spoliative* bleeding of ten or twelve ounces." (P. 163.)—If a thin patient is to be mentioned, "she offers exteriorly a considerable leanness." (P. 246.) "She had grown considerably leaner, which threw out into greater relief her meteorised belly." (P. 171.)—"M. Recamier relished highly the application of leeches." (P. 180.)—"The face was habitually easy to assume colour." (P. 194.) &c. &c. &c.

If Dr. Balbirnie's example be followed of translating literally from the French, the fears of our great lexicographer will be completely realized, and we shall certainly "babble a dialect of France," which, for a long time at least, the mere English reader will not comprehend. In addition to the specimens given above, we may state that Dr. B. rarely speaks of *symptoms;* he prefers "accidents." *Tissues* are always written "tissus." For the mucous membrane of the uterus, &c. he writes "the mucus," adapting the French idiom, and remaining unintelligible to the English reader. "A brush of charpy" is an often-repeated expression. "Vulve" is always preferred to vulva. "Sophy S. *contraried* in her first inclinations." Lastly, we assure Dr. Balbirnie that the term "diet" does not inform the English student whether the patient was allowed as much meat as she could eat, or whether she was confined to bread and water. From almost every page of the book we might take similar specimens of Dr. B.'s very singular style of writing, and of the transference of French idioms into English words, to the destruction of his own language. We seriously, and certainly in a friendly spirit, recommend him to study a little more than he has hitherto done the good models in his own language, before he ventures to the press with the other works which he announces; and we have no doubt, from the industry and professional information he evidently possesses, that he may then offer "an acceptable text-book to students," which the volume we have noticed was intended to be.

---

